# Dairy intake in association with asthma symptoms among a large sample of children and adolescents: a cross-sectional study

**DOI:** 10.3389/fnut.2024.1298704

**Published:** 2024-03-21

**Authors:** Mona Jamalvandi, Bahareh Sasanfar, Zahra Nafei, Nasrin Behniafard, Marjan Jafari, Amin Salehi-Abargouei

**Affiliations:** ^1^Research Center for Food Hygiene and Safety, School of Public Health, Shahid Sadoughi University of Medical Sciences, Yazd, Iran; ^2^Department of Nutrition, School of Public Health, Shahid Sadoughi University of Medical Sciences, Yazd, Iran; ^3^Student Research Committee, Shahid Sadoughi University of Medical Sciences, Yazd, Iran; ^4^Cancer Research Center, Cancer Institute of Iran, Tehran University of Medical Sciences, Tehran, Iran; ^5^Children Growth Disorder Research Center, Shahid Sadoughi University of Medical Sciences, Yazd, Iran; ^6^Department of Allergy and Clinical Immunology, Shahid Sadoughi University of Medical Sciences, Yazd, Iran; ^7^Yazd Cardiovascular Research Center, Non-Communicable Diseases Research Institute, Shahid Sadoughi University of Medical Sciences, Yazd, Iran

**Keywords:** dairy intake, milk, asthma, wheezing, children, adolescents

## Abstract

**Background and objective:**

Dairy products may be associated with an increased risk of asthma, although there is little scientific evidence to support this association. The goal of this study was to explore the association between dairy consumption and asthma symptoms.

**Materials and methods:**

This cross-sectional study was conducted on children and adolescents aged 6–8 and 13–14 years living in central Iran. Dietary food consumption was assessed using a multiple-choice questionnaire. Logistic regression was used to obtain odds ratios for the association between milk, other dairy products, and total dairy consumption with the risk of asthma symptoms.

**Results:**

In total, 7,667 participants (3,414 boys and 4,253 girls) were included in the current study. Milk intake and total dairy consumption were not associated with the likelihood of wheezing, asthma confirmed by a doctor, current asthma, and asthma medication use. In addition, there was no association between other dairy product intake and the odds of wheezing in the past 12 months in the crude model. However, after adjusting for several confounders, those in the top category had lower odds of wheezing in the past 12 months than those in the bottom category (OR: 0.58; 95% CI: 0.40–0.85).

**Conclusion:**

The consumption of dairy products other than milk, including cheese and yogurt, might reduce the likelihood of wheezing in children and adolescents.

## Introduction

Asthma is the most common chronic condition in children and adolescents (1), affecting approximately 6.6 million children in the United States (2). Exacerbations of asthma are a leading cause of disease morbidity, higher healthcare expenses, and, in some individuals, a faster loss of lung function (3). Wheezing, coughing, shortness of breath, and chest tightness are some of the symptoms that might occur (4). In 1980, the prevalence of asthma was accounted to be approximately 3.5% among children. However, the number has grown to 9.5% in approximately 30 years (5). Adolescents have the same or higher prevalence and morbidity from asthma than the rates in children (6). In 2010, this disease in white Americans (7.8%) is less common than African Americans (11.9%) (5), and the highest prevalence rates are reported in developed countries like Australia (21%). In total, 27 papers reported that the prevalence rate of “asthma ever” among Iranian children ranged from 0.5 to 11.0% (4). A meta-analysis of 97,205 subjects aged 1–16 years showed that the overall prevalence of asthma in children was 5.1% (7). According to two consecutive reports from the International Study of Asthma and Allergies in Childhood (ISAAC) in 1998 and 2004, the prevalence of asthma among Iranian children aged 13–14 years increased from 10.9 to 13.2% in one period. This finding indicates a rising trend of asthma in this age group (8). The 6-year rate of asthma prevalence among Iranian children aged 13–14 years was 2.3%, and the annual increase was 0.17%. However, the global prevalence of asthma symptoms in children aged 13–14 years from 1998 to 2004 was only 0.13%. This finding suggests that Iran has a higher and faster rate of asthma than the world average in this age group (9–11).

The causes of asthma are not fully understood. However, several risk factors, including microbiome and viral infections, smoking (12), vitamin D status, chemical exposure, air pollution (13), pollen (14), stress, genetic factors (15), and dietary changes (16) are proposed to be associated with the development of the disease. The relationship between nutrients, foods, food groups, dairy products, vitamin D3 (17), antioxidants (18), soy isoflavones (19), and fish oil (20) and asthma has been explored by several studies (13, 21). Although dairy products are high in micronutrients, fatty acids, and probiotics, it is believed that they may raise the incidence of asthma and allergies in children (22). It is proposed that dairy products could flare up asthma symptoms, although there is little scientific evidence to support this theory (23). In this regard, the results of epidemiological studies are inconsistent. Several studies have found a strong association between dairy consumption and asthma, wheezing, eczema, and rhinitis in children (24, 25), whereas a null association has been found in another study of children and adults (26–32). For example, a study in Greece showed that consumption of farm milk at the age of 5 years is associated with a lower prevalence of atopy at the age of 11–19 years (33). A case–control study published in 2015 found that increased consumption of dairy products was significantly associated with higher odds of asthma (34). In another study, whole milk and butter were shown to be negatively associated with asthma, whereas the consumption of soy beverage, ricotta cheese, and low-fat cheese were shown to be positively associated with asthma (26). However, a double-blind, placebo-controlled, crossover study could not confirm that dairy products might induce bronchoconstriction or changes in asthma symptoms in adults (23). According to previous studies, more than half of the people with asthma change their eating habits, with dairy products being the most typically avoided food (26).

To our knowledge, data on dairy intake and asthma association are limited in the Middle Eastern countries, and most of the studies were conducted in the developed countries (35). It is noteworthy that the dietary habits of the Middle East have unique characteristics that contain high amounts of fruits and vegetables, rich sources of antioxidants, and large amounts of refined carbohydrates and detrimental fats. Therefore, the present study aimed to investigate the association between the consumption of dietary milk and other dairy products and asthma and its symptoms in a large sample of children and adolescents in Iran.

## Subjects and methods

### Participants

The current cross-sectional study was part of the Global Asthma Network (GAN), which took place in Yazd, central Iran, in early 2020. The GAN is a cross-sectional, multicenter, multicountry epidemiological study that builds on and enhances the ISAAC Phase Three methodology (36). According to the GAN recommendation, at least 3,000 samples are required to estimate a good prevalence of asthma (37). In the present study, students from 48 and 36 high and elementary schools (state and private), respectively, were randomly selected from two educational districts using a cluster sampling design, respectively. Moreover, non-Iranian students were excluded from the study. All the subjects aged 13–14 years and parents of 6–7 years were invited to fill out online electronic questionnaires on asthma and its symptoms and risk factors, which were designed and placed in the virtual education groups of schools. The information was collected using paper questionnaires for a group of participants aged 6–8 years. Due to the school closures during the COVID-19 pandemic, the rest of the 6- to 8-year-old students were provided with the electronic questionnaires. Out of 7,214 and 3,026 adolescents and children, 5,141 and 2,526 of them, respectively, completed the questionnaire, with response rates of 71.3 and 83.5%, respectively, and then, demographic data that seemed unacceptable were reviewed by telephone and corrected if necessary. At the beginning of the study, due to schools’ closure during the outbreak of COVID-19, an electronic questionnaire was designed and placed in the virtual education groups of schools.

The ethics committee of Shahid Sadoughi University (SSU) of Medical Sciences in Yazd, Iran, approved the GAN study on Iranian youngsters (IR.SSU.REC.1398.244). The ethics committee also gave its approval for the current study. The Yazd Education administration then granted permission to conduct the study at elementary and guidance schools. Parents gave their informed consent as well. The consent form was included at the start of the internet questionnaires so that children and their parents may feel entirely comfortable participating in the research.

### Confirmation of asthma and its symptoms

The GAN questionnaire, derived from the ISAAC questionnaire (29), includes questions about the symptoms of allergic diseases and related risk factors. In this study, we used some questions about asthma symptoms, “use of asthma medication” and “asthma confirmed by a doctor,” and the amount of dairy consumed in the diet last year. According to the protocol of this study, current asthma was defined as a history of confirmed asthma by a doctor and having had wheezing and/or use of asthma medication in the past 12 months. Once the questionnaire was translated into Persian, the reliability of the translated version was confirmed by a study conducted on 100 selected subjects using Cronbach’s alpha. The alpha coefficient for asthma symptoms was estimated to be 0.862, thus exhibiting appropriate internal consistency. Finally, the questionnaire was translated back into English and sent to the GAN principals in order to be approved.

### Assessment of dietary intake

In this study, the dietary intake of children’s food groups in the last 12 months was assessed using one of the multiple-choice questions in the GAN questionnaire (38). The frequency response section had three options (never or occasionally/once or twice a week most/ all days of the week). Milk, other dairy products (yogurt, cheese, cream, top milk, and kashk), and total dairy consumption were assessed. The frequency of total dairy intake was assessed by summing dietary milk (including flavored milk), cheese, and yogurt consumption.

### Assessment of other variables

The data on participants’ height, weight, ethnicity (Kord/Turk/Persian/Lor/Arab/Balooch), watching television, and computer use (2–4 h/5–8 h/9–14 h a day) were obtained using a self-reported online GAN questionnaire. The body mass index (BMI) was calculated by using the following formula: weight (kg) divided by height squared (m^2^).

### Statistical methods

We used STATA version 14 (State Crop., College Station, TX) for all analyses. To compare continuous and categorical variables between individuals with and without “asthma confirmed by a doctor” and “usage of asthma medication,” independent samples of Student’s *t*-tests and the chi-squared test were used, respectively. A multivariable logistic regression analysis was used in the crude and multivariable controlled models to investigate the relationship between dairy intake and the odds of asthma confirmed by a doctor, current asthma, usage of asthma medication, and wheezing in the last 12 months. In the first model, adjustments were made for age and sex. Additional adjustments were made for watching TV and computer use in the second model. In the last model (third model), an additional adjustment for BMI was made. In all models, the lowest level of dairy intake (never or only occasionally) was considered as the reference category. A *p*-value of <0.05 was considered as statistically significant.

## Results

This study included 7,667 participants in total (3,414 boys and 4,253 girls). Table 1 shows that, according to sex, there were significant differences between the people with and without asthma confirmed by a doctor and between people with and without the use of asthma medication. In addition, based on wheezing in the past 12 months, a significant difference was observe between the same factors (*p* < 0.001). In terms of ethnicity, a significant difference was observed between the subjects with and without the use of asthma medication (*p* < 0.01). Individuals with asthma confirmed by a doctor and with the use of asthma medication were older compared with those without these factors (10.9 vs. 11.7, *p* < 0.001) (10.9 vs. 11.3, *p* = 0.05).

**Table 1:** General characteristics of the subjects according to asthma.

Variables	Asthma confirmed by a doctor		*p*-value	Use of asthma medication		*p*-value
	Without (*n* = 7,343)	With (*n* = 324)		Without (*n* = 7,476)	With (*n* = 191)	
Sex						
Male	3,226 (43.93)	188 (58.02)	<0.001	3,296 (44.09)	118 (61.7)	<0.001
Female	4,117 (56.07)	136 (41.98)		4,180 (55.9)	73 (38.2)	
Age (years)	10.9 ± 3.37	11.7 ± 2.94	<0.001	10.9 ± 3.36	11.3 ± 3.16	0.05
BMI (kg/m^2^)	18.9 ± 10.4	19.1 ± 4.18	0.35	18.97 ± 10.3	18.73 ± 3.97	0.37
Ethnicity	0.003
Kord		38 (0.52)	5 (1.54)	0.13	38 (0.51)	5 (2.62)
Turk		73 (0.99)	2 (0.62)	74 (0.99)	1 (0.52)
Persian		7,064 (96.2)	311 (95.9)	7,192 (96.2)	183 (95.8)
Lor		62 (0.84)	4 (1.2)	64 (0.86)	2 (1.05)
Arab		55 (0.75)	1 (0.31)	56 (0.75)	0 (0.0)
Balooch		51 (0.69)	1 (0.31)	52 (0.70)	0 (0.0)
Watching television and computer use	0.29
2–4 h		3,945 (53.7)	163 (50.3)	0.38	4,016 (53.7)	92 (48.1)
5–8 h		2,463 (33.5)	103 (34.8)	2,506 (33.5)	70 (36.6)
9–14 h		935 (12.7)	48 (14.8)	954 (12.7)	29 (15.1)
Wheezing (in the past 12 months)
Yes	553 (7.5)	56 (17.2)	<0.001	518 (6.9)	91 (47.6)	<0.001
No	6,790 (92.4)	268 (82.7)		6,958 (93.0)	100 (52.3)	
Milk intake
Never	1,282 (17.4)	51 (15.7)	0.71	1,300 (17.3)	33 (17.2)	0.21
Weekly	3,854 (52.4)	175 (54.0)		3,939 (52.6)	90 (47.1)	
Every day	2,207 (7343)	98 (30.2)		2,237 (29.9)	68 (35.6)	
Other dairy intake
Never	286 (3.8)	10 (3.09)	0.15	287 (3.8)	9 (4.7)	0.16
Weekly	2,273 (30.9)	86 (26.5)		2,312 (30.9)	47 (24.6)	
Every day	4,784 (65.1)	228 (70.3)		4,877 (65.2)	135 (70.6)	
Total Dairy intake
Never	178 (2.4)	7 (2.1)	0.21	178 (2.3)	7 (3.6)	0.19
Weekly	2061 (28.0)	77 (23.7)		2094 (28.0)	44 (23.0)	
Every day	5,104 (69.5)	240 (74.0)		5,204 (69.6)	140 (73.3)	

Values are mean (SD) or percentages.

^a^*χ*^2^ Test for ordinal qualitative variables and *t*-test for continuous variables.

Tables 2–4 show the multivariable-adjusted odds ratios and 95% confidence intervals for asthma confirmed by a doctor, current asthma, and use of asthma medication in the past 12 months across categories of dietary milk, other dairy, and total dairy consumption. According to the findings, there is no association between dairy consumption and doctor-diagnosed asthma, current asthma, or asthma medication use.

**Table 2:** The association between dairy intake and the likelihood of asthma confirmed by a doctor.

	Crude	Model 1	Model 2	Model 3
	0R (95% CI)	0R (95% CI)	0R (95% CI)	0R (95% CI)
Milk				
Never or only occasionally	1.00	1.00	1.00	1.00
Once or twice per week	0.97 (0.71–1.32)	0.95 (0.69–1.29)	0.95 (0.70–1.29)	0.95 (0.70–1.30)
Most or all days	1.00 (0.72–1.40)	0.98 (0.70–1.37)	0.98 (0.70–1.37)	0.99 (0.70–1.38)
P _trend_	0.93	0.99	0.99	0.98
Other dairy				
Never or only occasionally	1.00	1.00	1.00	1.00
Once or twice per week	1.57 (0.75–3.27)	1.51 (0.72–3.14)	1.51 (0.72–3.14)	1.51 (0.72–3.14)
Most or all days	1.62 (0.79–3.33)	1.45 (0.71–2.98)	1.46 (0.71–2.99)	1.46 (0.71–2.99)
P _trend_	0.32	0.70	0.70	0.69
Total dairy				
Never or only occasionally	1.00	1.00	1.00	1.00
Once or twice per week	1.26 (0.54–2.93)	1.19 (0.51–2.76)	1.18 (0.51–2.76)	1.18 (0.50–2.75)
Most or all days	1.34 (0.59–3.07)	1.18 (0.52–2.71)	1.18 (0.51–2.71)	1.18 (0.51–2.70)
P _trend_	0.44	0.84	0.85	0.84

Model 1: adjusted for age and sex.

Model 2: further adjusted for watching TV and computer use.

Model 3: additionally, adjustment for BMI.

**Table 3:** The association between dairy intake and the likelihood of current asthma.

	Crude	Model 1	Model 2	Model 3
	0R (95% CI)	0R (95% CI)	0R (95% CI)	0R (95% CI)
Milk				
Never or only occasionally	1.00	1.00	1.00	1.00
Once or twice per week	0.91 (0.44–1.89)	0.93 (0.45–1.93)	0.93 (0.45–1.93)	0.93 (0.45–1.93)
Most or all days	1.03 (0.47–2.24)	0.98 (0.45–2.15)	0.98 (0.45–2.15)	0.99 (0.45–2.15)
P _trend_	0.86	0.98	0.98	0.98
Other dairy				
Never or only occasionally	1.00	1.00	1.00	1.00
Once or twice per week	0.81 (0.24–2.76)	0.81 (0.24–2.77)	0.81 (0.24–2.77)	0.81 (0.24–2.77)
Most or all days	0.63 (0.19–2.09)	0.70 (0.21–2.32)	0.70 (0.21–2.32)	0.70 (0.21–2.32)
P _trend_	0.28	0.47	0.47	0.47
Total dairy				
Never or only occasionally	1.00	1.00	1.00	1.00
Once or twice per week	0.45 (0.13–1.59)	0.47 (0.13–1.65)	0.47 (0.13–1.65)	0.47 (0.13–1.65)
Most or all days	0.42 (0.12–1.39)	0.47 (0.14–1.57)	0.47 (0.14–1.57)	0.47 (0.14–1.57)
P _trend_	0.34	0.52	0.52	0.52

Model 1: adjusted for age and sex.

Model 2: further adjusted for watching TV and computer use.

Model 3: additionally, adjustment for BMI.

**Table 4:** The association between dairy intake and the use of asthma medication.

	Crude	Model 1	Model 2	Model 3
	0R (95% CI)	0R (95% CI)	0R (95% CI)	0R (95% CI)
Milk				
Never or only occasionally	1.00	1.00	1.00	1.00
Once or twice per week	0.90 (0.60–1.34)	0.87 (0.58–1.30)	0.87 (0.58–1.30)	0.87 (0.58–1.31)
Most or all days	1.19 (0.78–1.82)	1.12 (0.73–1.71)	1.12 (0.73–1.71)	1.12 (0.73–1.72)
P _trend_	0.24	0.38	0.38	0.37
Other dairy products				
Never or only occasionally	1.00	1.00	1.00	1.00
Once or twice per week	0.64 (0.31–1.33)	0.60 (0.29–1.25)	0.61 (0.29–1.26)	0.60 (0.29–1.25)
Most or all days	0.88 (0.44–1.75)	0.78 (0.39–1.56)	0.79 (0.39–1.57)	0.78 (0.39–1.56)
P _trend_	0.26	0.46	0.47	0.49
Total dairy				
Never or only occasionally	1.00	1.00	1.00	1.00
Once or twice per week	0.53 (0.23–1.20)	0.49 (0.22–1.12)	0.49 (0.21–1.12)	0.50 (0.22–1.13)
Most or all days	0.68 (0.31–1.48)	0.60 (0.27–1.31)	0.59 (0.27–1.30)	0.59 (0.27–1.30)
P _trend_	0.52	0.83	0.85	0.87

Model 1: adjusted for age and sex.

Model 2: further adjusted for watching TV and computer use.

Model 3: additionally, adjustment for BMI.

Table 5 shows the multivariable-adjusted odds ratios and 95% confidence intervals for wheezing in the previous 12 months by dairy consumption category. There was no association between milk and total dairy intake and the odds of wheezing in the past 12 months. In addition, there was no association between the intake of other dairy products and the odds of wheezing in the past 12 months in the crude model. However, after adjusting for several confounders, those in the top category had lower odds of wheezing in the past 12 months than those in the bottom category (OR: 0.58; 95% CI: 0.40–0.85).

**Table 5:** The association between dairy intake and the likelihood of wheezing in the past 12 months.

	Crude	Model 1	Model 2	Model 3
	0R (95% CI)	0R (95% CI)	0R (95% CI)	0R (95% CI)
Milk				
Never or only occasionally	1.00	1.00	1.00	1.00
Once or twice per week	0.89 (0.71–1.11)	0.87 (0.69–1.09)	0.88 (0.70–1.10)	0.88 (0.70–1.10)
Most or all days	0.92 (0.72–1.18)	0.90 (0.70–1.15)	0.91 (0.71–1.17)	0.91 (0.71–1.17)
P _trend_	0.65	0.54	0.60	0.61
Other dairy products				
Never or only occasionally	1.00	1.00	1.00	1.00
Once or twice per week	0.65 (0.44–0.96)	0.62 (0.42–0.91)	0.63 (0.42–0.93)	0.63 (0.42–0.93)
Most or all days	0.65 (0.44–0.94)	0.58 (0.39–0.84)	0.58 (0.40–0.85)	0.58 (0.40–0.85)
P _trend_	0.18	**0.03**	**0.02**	**0.02**
Total dairy				
Never or only occasionally	1.00	1.00	1.00	1.00
Once or twice per week	0.76 (0.46–1.25)	0.71 (0.43–1.18)	0.70 (0.42–1.16)	0.70 (0.42–1.16)
Most or all days	0.74 (0.45–1.20)	0.65 (0.40–106)	0.63 (0.38–1.03)	0.63 (0.38–1.03)
P _trend_	0.37	0.09	0.07	0.07

Model 1: adjusted for age and sex.

Model 2: further adjusted for watching TV and computer use.

Model 3: additionally, adjustment for BMI.

*p*-trend < 0.05 (they are statistically significant) are bolded.

## Discussion

According to this study, higher consumption of other dairy products (such as cheese or yogurt) was related to a lower risk of wheezing in the previous 12 months. To our knowledge, this study is among the first studies that report the association between dairy intake and the odds of asthma symptoms in a Middle Eastern country.

We found a significant inverse association between other dairy products and the risk of wheezing in the previous 12 months. Similar to our results, a cross-sectional study of 1,601 participants in Melbourne showed a negative association between dairy product consumption and risk of asthma (26). In a cross-sectional study of 1,444 children aged 12 years, Hijazi et al. assessed the relationship between asthma and wheezing risk factors with diet through the ISAAC questionnaire and found that decreased milk consumption is associated with a higher prevalence of asthma symptoms and allergy symptoms (24). PIAMA birth cohort research in 2,978 children found that frequent use of milk fat-containing items was associated with a lower risk of asthma symptoms (13). It is noteworthy that eating dairy products (even a few times a week) had an inversely significant influence on asthma compared to never eating them (39). According to data of Wijga et al., 2,978 children aged 2–3 years participated, and their data were collected by food frequency data; it showed that consumption of milk fat reduced symptoms of asthma (13). In a study by Riedler et al. on 3,504 children aged 6–13 years, after completing the questionnaire on asthma, hay fever, and atopic sensitization, serum IgE antibodies was assessed. Consumption of farm milk aged 1–5 years was associated with lower asthma frequency than children younger than 1 year. Thus, long consumption of farm milk has effects of protection against asthma and its symptoms (40). In contrast, Malaeb et al. found that consuming less milk on a regular basis reduced the current asthma risk (41). In the same direction, based on the Chinese study, with the aim of association between food groups and allergic disease in school children (7–12 years), high milk consumption was positively linked with asthma (42). According to a systematic review and meta-analysis in 2023, with the title of association milk and dairy product consumption and asthma risk in children, negative association between the consumption of milk and dairy products and asthma was not seen (35). Another cross-sectional study in 1,014 students (5–14 years old) illustrated that respiratory symptoms were stronger among those who consumed less fresh milk and milk fat and more margarine (25). A cohort study in Japan showed that higher maternal intake of total dairy products, cheese, yogurt, and calcium during pregnancy may reduce the risk of infantile eczema in the previous 12 months, physician-diagnosed asthma, and physician-diagnosed atopic eczema (43). However, Tabak et al. found no apparent relation between dairy products and asthma (30). In another study, the effect of milk consumption on the risk for asthma in 246 girls at the age of 8–10 years was investigated. Data on the consumption of milk in the last year were collected by questionnaires. Based on the findings of this study, rare milk consumption was associated with asthma risk in girls (44).

Milk and dairy products are high in saturated fat, which has been related to a lower incidence of allergy disorders and asthma (41). Butter and whole milk consumption may be beneficial for asthma and allergy symptoms. However, margarine consumption was found to be detrimental to asthma and allergy symptoms (45). The beneficial effect of milk and butter may stem from the fatty acid of milk (45), and in addition to the fact that the digestion of lactose is different from other carbohydrates, it may act as a conditional prebiotic (46). The prebiotic activity of dairy products probably results from the stimulation of the growth of beneficial bacteria in the intestines, which may modulate immune responses and thus protect humans from asthma and allergies (47). Milk proteins, including lactalbumin, lactoglobulin, and immunoglobulins, whey proteins such as serum albumin, lactoferrin, and lactoperoxidase, and various enzymes and cytokines found in dairy products are believed to be responsible for this protective effect (48). It should be noticed that, in our study, 7.5% of the subjects experienced wheezing in the past 12 months without asthma confirmed by a doctor. This could be attributed to the onset of symptoms at an older age or the possibility that some individuals developed wheezing after contracting a respiratory illness such as COVID-19 or the flu. On the other hand, 83% of the patients diagnosed with asthma by a doctor did not experience wheezing in the last 12 months, which may be due to either an inaccurate diagnosis of asthma in these children or the effective management of the condition, resulting in the absence of symptoms.

Many researchers have looked at the medicinal and preventative effects of yogurt and the lactic acid bacteria that are widely utilized in yogurt production (49). The relationship between cytokine imbalance and asthma symptoms has attracted attention. A shift away from a TH1 interferon-gamma (IFN-γ) pattern toward a TH2 (IL-4, IL-5, and IL-13) profile is noticed in atopic illness immunological responses. Interferon-gamma is a lymphokine that activates macrophages. Incomplete IFN-γ production, according to these findings, predisposes to the development of allergy disorders and asthma (50). Human studies showed that a long-term consumption of substantial amounts of yogurt (450 g/day) resulted in boosting the production of IFN-γ by lymphocytes and separated T cells. Oral ingestion of *Lactobacillus casei* and other probiotic strains (51) has been shown to reduce immunoglobulin E (IgE) production. These findings suggest that eating yogurt could help reduce IgE-mediated diseases, including asthma (49). Conjugated linoleic acid (CLA) is one of the other probable pathways discussed in previous studies. These compounds have a wide range of biological features that could help asthma sufferers, including impacts on energy management, lipid metabolism, inflammation, and immunological function. Metabolic benefits of CLA, which include fat loss and adipokine regulation, may be beneficial for respiratory mechanics and systemic inflammation, which may apply to asthmatic airway inflammation (52).

The present study has considerable strengths. A high sample size reduces the risk of selection bias, and because the study was conducted on a general population, it included different ethnicities. The current study includes various flaws that should be taken into account when evaluating the results. The current study relied solely on self-reported data, with questions about milk and other dairy products included in the questionnaire. Furthermore, although the associations were adjusted for several possible confounding variables, we had no data on some other variables, including energy intake, physical activity, and other variables. We did not collect data on the type of schools (public vs. private). Additionally, information about environmental and eating habits in different ethnicities, other than milk/dairy product consumption, was not available. It is also possible that how milk is consumed (crude vs. pasteurized) varies among different ethnicities. Therefore, residual confounding might be a limitation of the current study. As each dairy product intake was not asked in the questionnaire, we were unable to check the association for each dairy product. In cross-sectional studies, the dependent and independent variables were gathered at the same time; therefore, no causal association can be inferred from their results. Another important limitation of the study is that conclusions can only be drawn regarding the inverse relation between the high frequency of dairy products consumption and wheezing in the previous 12 months. Wheezing can be caused by various diseases, such as gastroesophageal reflux, recurrent viral respiratory tract infections, cystic fibrosis, bronchopulmonary dysplasia, persistent bacterial bronchitis, immune deficiency, primary ciliary dyskinesia, congenital heart disease, and tuberculosis (53, 54). Moreover, it cannot be concluded that consumption of dairy products necessarily leads to protection against asthma. Therefore, future prospective studies are highly recommended.

## Conclusion

In conclusion, the current investigation showed that most or daily intake of cheese, yogurt, cream, or kashk among children and adolescents was associated with 42% lower odds of wheezing in the past 12 months compared with those with never/or occasional intake. These findings will need to be confirmed in prospective cohort studies.

## Data availability statement

The original contributions presented in the study are included in the article/supplementary material, further inquiries can be directed to the corresponding author.

## Ethics statement

The studies involving humans were approved by the GAN study on Iranian youngsters (IR.SSU.REC.1398.244). The studies were conducted in accordance with the local legislation and institutional requirements. Written informed consent for participation in this study was provided by the participants’ legal guardians/next of kin.

## Author contributions

MoJ: Formal analysis, Writing – original draft. BS: Formal analysis, Writing – original draft. ZN: Conceptualization, Writing – review & editing. NB: Conceptualization, Supervision, Writing – review & editing. MaJ: Data curation, Writing – original draft. AS-A: Supervision, Writing – review & editing, Supervision, Writing – review & editing.
